# A MAGIC GATHERING: PILOTING AN AUGMENTED REALITY ENGINE TO PROMOTE MENTAL HEALTH AND WELL-BEING

**DOI:** 10.1093/geroni/igz038.1209

**Published:** 2019-11-08

**Authors:** Thomas Chan, Daniel Matook, Isaac Argueta, Jonathan Kennedy, Jeremy Argueta, Thomas Chan

**Affiliations:** California State University Northridge, Northridge, California, United States

## Abstract

Augmented reality (AR), superimposing digital assets onto the real-world (i.e., holograms)—offers a paradigm shift in delivering immersive interventions for aging adults. Reminiscence and life review are robust intervention techniques that have decades of empirical support to boost mood and meaning in older adults. In this presentation, we demonstrate the applied research and technology development process to create an AR reminiscence and life review engine for aging adults and their caretakers. We conducted 25 needs assessment interviews with community-dwelling older adults, and assisted living facility directors, nurses, and residents. Initial analyses revealed the need for: (1) finding scalable and cost-effective solutions to alleviate time burden for caregivers; (2) increasing variety of activities that do not need much instruction; (3) producing activities that grab and maintain attention; and, (4) generating more personalized activities that do not divert too much time from caretakers. We integrated the findings to develop a working prototype AR engine called “Project Phonado.” From preliminary user testing, Project Phonado aids in boosting mental health and meaning in aging adults—at least temporarily (immediate and one day follow-up). We will show clips of some pilot user testing of the engine and discuss the next evaluation and development steps.

